# Tri­fluoro­methane­sulfonate salt of 5,10,15,20-tetra­kis­(1-benzyl­pyridin-1-ium-4-yl)-21*H*,23*H*-porphyrin and its Ca^II^ complex

**DOI:** 10.1107/S205698902400447X

**Published:** 2024-05-21

**Authors:** María K. Salomón-Flores, Josue Valdes-García, Diego Martínez-Otero, Alejandro Dorazco-González

**Affiliations:** a Instituto de Química, Universidad Nacional Autónoma de México, Circuito Exterior, Ciudad Universitaria, Mexico, 04510, D.F., Mexico; b Centro Conjunto de Investigación en Química Sustentable, UAEM-UNAM, Instituto de Química, Universidad Nacional Autónoma de México, Carretera Toluca-Atlacomulco Km 14.5, CP 50200 Toluca, Estado de México, Mexico; Universidad de la Repüblica, Uruguay

**Keywords:** crystal structure: porphyrin, hydrogen bond, calcium complex,

## Abstract

The synthesis, crystallization and characterization of a tri­fluoro­methane­sulfonate salt of 5,10,15,20-tetra­kis­(1-benzyl­pyridin-1-ium-4-yl)-21*H*,23*H*-por­phy­rin, C_68_H_54_N_8_^4+^·4CF_3_SO_3_^−^·4H_2_O, **1**·OTf, are reported in this work. The reaction between 5,10,15,20-tetra­kis­(pyridin-4-yl)-21*H*,23*H*-porphyrin and benzyl bromide in the presence of 0.1 equiv. of Ca(OH)_2_ in CH_3_CN under reflux with an N_2_ atmosphere and subsequent treatment with silver tri­fluoro­methane­sulfonate (AgOTf) salt produced a red–brown solution. This reaction mixture was filtered and the solvent was allowed to evaporate at room temperature for 3 d to give **1**·OTf. Crystal structure determination by single-crystal X-ray diffraction (SCXD) revealed that **1**·OTf crystallizes in the space group *P*2_1_/*c*. The asymmetric unit contains half a porphyrin mol­ecule, two tri­fluoro­methane­sulfonate anions and two water mol­ecules of crystallization. The macrocycle of tetra­pyrrole moieties is planar and unexpectedly it has coordinated Ca^II^ ions in occupational disorder. This Ca^II^ ion has only 10% occupancy (C_72_H_61.80_Ca_0.10_F_12_N_8_O_16_S_4_). The pyridinium rings bonded to methyl­ene groups from porphyrin are located in two different arrangements in almost orthogonal positions between the plane formed by the porphyrin and the pyridinium rings. The crystal structure features cation⋯π inter­actions between the Ca^II^ atom and the π-system of the phenyl ring of neighboring mol­ecules. Both tri­fluoro­methane­sulfonate anions are found at the periphery of **1**, forming hydrogen bonds with water mol­ecules.

## Abstract 2

The synthesis and crystallization of a tri­fluoro­methane­sulfonate salt of 5,10,15,20-tetra­kis­(1-benzyl­pyridin-1-ium-4-yl)-21*H*,23*H*-porphyrin, C_68_H_54_N_8_^4+^·4CF_3_O_3_S·4H_2_O, **1**·OTf, are reported. The asymmetric unit contains half a porphyrin mol­ecule, two tri­fluoro­methane­sulfonate anions and two water mol­ecules of crystallization. The macrocycle of tetra­pyrrole moieties is planar and unexpectedly it has coordinated Ca^II^ ions in occupational disorder. This Ca^II^ ion has only 10% occupancy (C_72_H_61.80_Ca_0.10_F_12_N_8_O_16_S_4_).

## 1. Chemical context

Porphyrins are heterocyclic organic macrocycles; they are composed of four pyrrole subunits inter­connected at their α-carbon atoms through methine bridges. (Lee *et al.*, 2018 ▸) The structure of porphyrin can be found in nature, such as in various types of chloro­phylls and hemes. Chloro­phylls play a fundamental role in photosynthesis as light-gathering antennas and as charge-separation reaction systems. Hemes are one of the key components of biocatalysts and oxygen carriers in the blood. Without porphyrins, there can be no life on earth. (Hiroto *et al.*, 2017 ▸) Porphyrin has an expanded electronic structure of 18 π-electrons; the resulting aromaticity gives rise to unique photophysical and semiconductor properties that make these compounds have a wide range of applications, which include artificial photosynthesis, catalysis, mol­ecular electronics, sensors, non-linear optics, and solar cells (Lee *et al.*, 2018 ▸; Cook *et al.*, 2017 ▸). They are also useful in medicine as photosensitizers in the photodynamic therapy of cancer (PDT) and in the treatment of some bacterial infections (Uttamlal & Sheila Holmes-Smith, 2008 ▸).

For the past ten years, the motif of tetra­pyridyl­porphyrin in its free base (TPyP) and metalated form (MTPyP) has been one of the most used basic components in building blocks in the design of structural solids based on porphyrins in materials chemistry since it has a flat and rigid structure, bearing laterally divergent pyridyl groups prone to supra­molecular inter­action with neighboring entities (Seidel *et al.*, 2011 ▸; Lipstman & Goldberg, 2009*a* ▸,*b* ▸, 2010 ▸; Koner & Goldberg, 2009 ▸). In this work, the periphery of tetra­pyridyl­porphyrin was modified by the benzyl­ation of the pyridyl groups, obtaining the tetra­cationic salt of triflate 5,10,15,20-tetra­kis­(1-benzyl­pyridin-1-ium-4-yl)-21*H*,23*H*-porphyrin, **1**·OTf, which was studied as a fluorescent chemosensor for iodide in pure water in its bromide salt form (**1**·Br) published in a previous work (Salomón-Flores *et al.*, 2019 ▸). In this paper, we describe the most important structural characteristics of the **1**·OTf crystal, which presents positional disorder, since 90% of the crystal is made up of free-base porphyrin while 10% of the crystal the tetra­pyrrolic nucleus is coordinated to a Ca^II^ ion. 
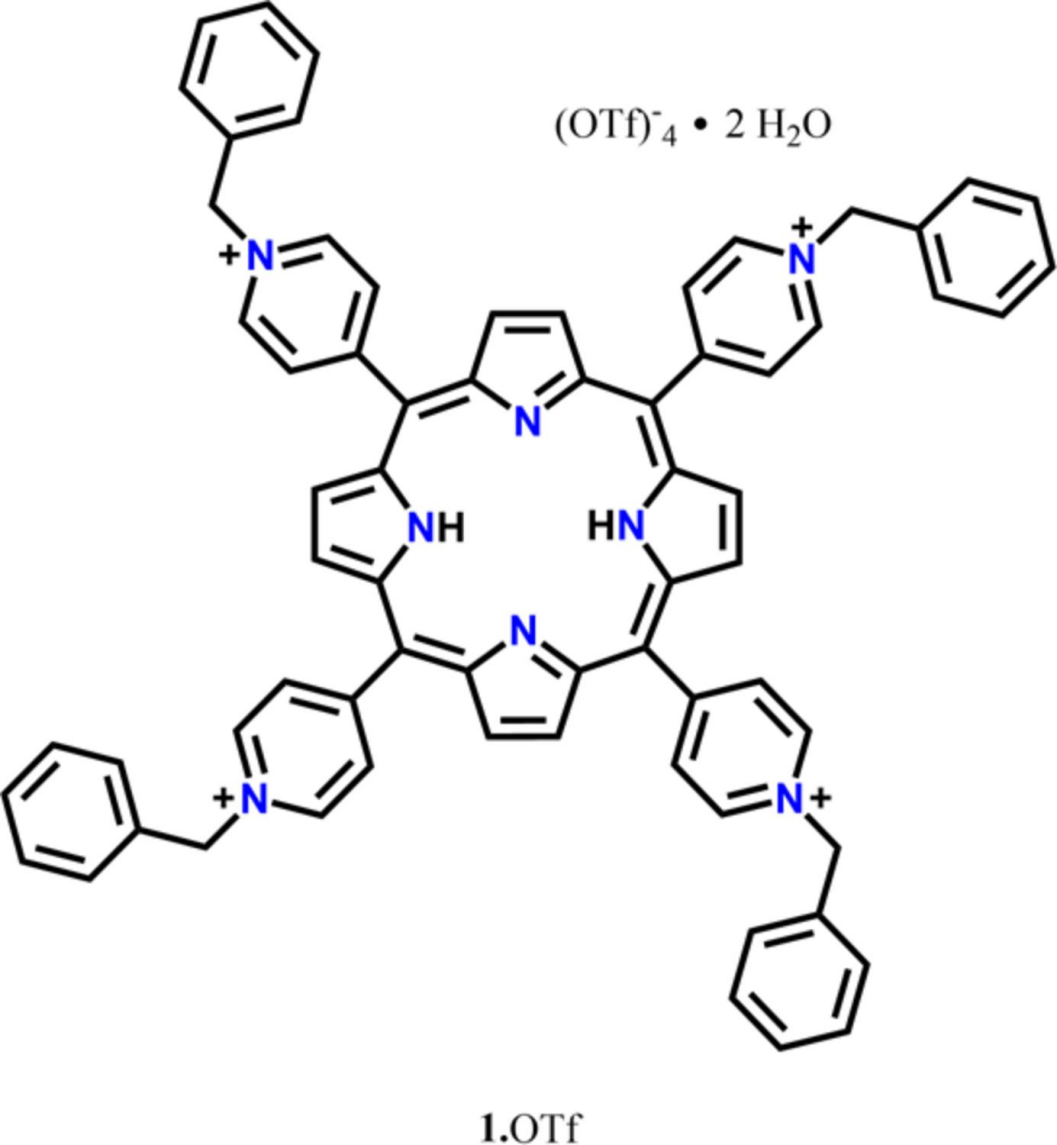


## 2. Structural commentary

**Figure 1:**
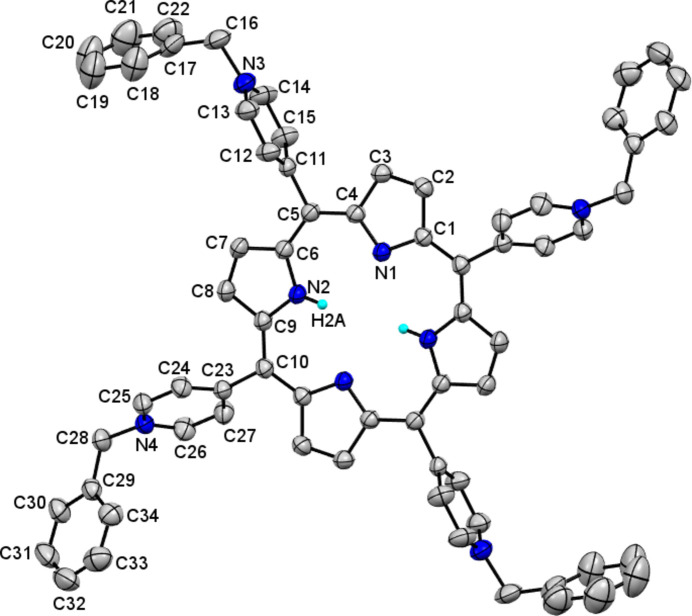
The mol­ecular structure of **1**·OTf. The atoms of the asymmetric unit are labeled. Hydrogen atoms, except for the –NH pyrroles, have been omitted for clarity.

Compound **1**·OTf crystallizes in the monoclinic system in space group *P*2_1_/*c* (Fig. 1 ▸). The asymmetric unit consists of half the **1**·OTf mol­ecule, two triflate anions to neutralize the charge, and two water mol­ecules of crystallization. The atoms of the triflate mol­ecules are in partially occupied sites. The degree of occupational disorder of the crystallization mol­ecules such as water, triflate and tosyl­ate are common in crystals of 5,10,15,20-tetra­kis (1-methyl­pyridinium-4-yl) cationic porphyrins (Lourenço *et al.*, 2011 ▸; Makowski *et al.*, 2012 ▸).

The C—C(*meso*), C—C and C—N bond lengths and angles in the pyrrole rings are in the ranges of 1.337 (2) to 1.451 (2) Å and 105.18 (12) to 126.89 (12)°, respectively, which are in the average ranges of bond lengths and angles reported for *meso*-pyridyl porphyrins. These macrocycle dimensions are relatively constant for all porphyrins, including complex multi-porphyrins, as well as the simpler derivatives of porphyrin (Konarev *et al.*, 2018 ▸; Cook *et al.*, 2017 ▸). Specifically, the C—N—C bond angles are 109.83 (12) and 105.18 (8)° for the nitro­gen atoms of the protonated and non-protonated pyrroles, respectively. The transannular separations N⋯N [N1⋯N1^i^ = 4.057 (2) Å and N2⋯N2^i^ = 4.186 (2) Å] are comparable with the values found in the bromide salt of 5,10,15,20-tetra­(benzyl­pyridinium)-21*H*,23*H* porphyrin **1**·Br (4.042 and 4.195 Å) and N⋯N distances between adjacent N atoms in **1**·OTf [2.887 (2) and 2.942 (2) Å] are also similar to those of **1**·Br (2.868 and 2.957 Å; Salomón-Flores *et al.*, 2019 ▸).

The tetra­pyrrole macrocycle is characteristically rigid and flat; the deviations of the individual atoms from the mean plane of the 24-membered porphyrin core range from 0.004 (1) (C1) to 0.060 (2) Å (C8), the core of **1**·OTf is flatter than **1**·Br, 100% free base, with values of atomic deviations from 0.012 (N1) to 0.094 Å (N2). The four pyridinium rings in the *meso* positions are in two different arrangements. The first pyridinium ring forms an almost orthogonal arrangement between the plane of the 24-membered porphyrin and the pyridinium ring N3/C11–C15 with an angle between the planes of 85.1 (3)°, while the second pyridinium ring N4/C23–C27 forms an angle between the planes of 61.54 (6)°. These angles are large due to the steric hindrance by the benzyl groups and their values are similar to those of **1**·Br of 81.3 and 57.3°. Likewise, the benzyl groups are almost perpendicular to the corresponding pyridiniums, the angles between their planes being 77.1 (3) and 84.32 (1)° for the two benzyl­pyridinium fragments. The dihedral angle between the adjacent pyrrole rings N1/C1–C4 and N2/C6–C9 is 4.90 (9)°. The planes of the pyrrole rings are inclined to the N_4_ plane by 3.03 (7)° (N1/C1–C4 ring) and 4.71 (7)° (N2/C6–C9 ring), therefore the overall degree of distortion of the macrocycle is moderate and there is also no significant effect of the benzyl groups on the planar geometry of the porphyrin.

**Figure 2:**
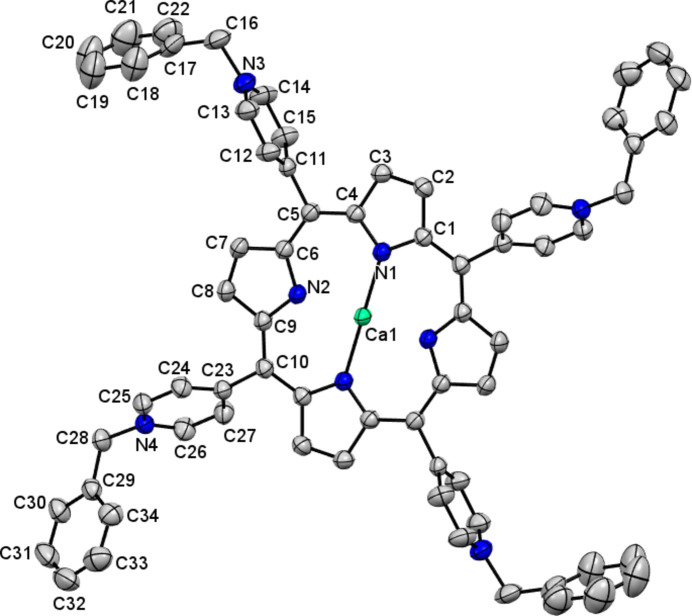
The mol­ecular structure of **1**·OTf-Ca. The atoms of the asymmetric unit are labeled. Hydrogen atoms have been omitted for clarity.

In this single crystal, one in ten entities of **1**·OTf, has a Ca^II^ ion coordinated in its tetra­pyrrolic nucleus; this cation presents an occupational disorder. Fig. 2 ▸ shows the mol­ecular structure of **1**·OTf-Ca. The calcium(II) atom coordinates to the four pyrrolic nitro­gen with a distorted square geometry and no ligand coordinated axially. In this context, complexes with high coordination numbers (hepta­coordinate) of Ca^II^ have been reported in porphyrins and porphyrinogens as well as with N-donor ligands (Bonomo *et al.*, 1999 ▸, 2001 ▸; Fromm, 2020 ▸; Dyall *et al.*, 2019 ▸). For example, the calcium atom in 5,10,15,20-tetra­kis­(4-tert-butyl­phen­yl)porphyrinato calcium(II) {[Ca(^*t*^BuPP)(Py)_3_]} is hepta­coordinated with three pyridines and Ca—N bond distances in the tetra­pyrrolic macrocycle [Ca—N = 2.382 (4) and 2.416 (3) Å] are larger than found for **1**·OTf-Ca [Ca1—N1 = 2.0284 (12) and Ca1—N2 = 2.0928 (12) Å] due to Ca(II) protruding from the N_4_ plane of the tetra­pyrrolic nucleus (Bonomo *et al.*, 2001 ▸). In the case of **1**·OTf-Ca, Ca^II^ is exactly coplanar to the tetra­pyrrolic plane and is not out of the N_4_ plane, in comparison to [Ca(tBuPP)(Py)_3_] that has a distance of 1.657 (5) Å. Furthermore, the N—Ca—N bond angles are 88.92 (5), 91.08 (5) and 180°.

## 3. Supra­molecular features

The porphyrin macrocycle of **1**·OTf presents π-electron deficiency as a result of the multiple positive charge of the *N*-benzyl­pyridinium groups and is stabilized mainly by electrostatic inter­actions with the triflate anions; however, other supra­molecular inter­actions also stabilize the crystal.

The *N*-benzyl­pyridinium groups in **1**·OTf produce steric hindrance, which prevents the aggregation of porphyrin mol­ecules and π–π stacking inter­actions between the tetra­pyrrolic nuclei, which are common in free-base tetra­pyridyl­porphyrins (Seidel *et al.*, 2011 ▸; Lipstman & Goldberg, 2009*b* ▸, 2010 ▸). Conversely, salts of tetra­pyridinium porphyrins quaternized with small groups such as –CH_3_ and –H generate porphyrin mol­ecules offset-stacked; this cofacial arrangement is a well-known feature of the supra­molecular inter­porphyrin organization. However, bulky groups as substituents in the *meso* positions of the porphyrins can hinder the inter­actions between porphyrins (Lourenço *et al.*, 2011 ▸; Makowski *et al.*, 2012 ▸; Wang *et al.*, 2013 ▸; Zhao *et al.*, 2013 ▸), as in this case.

**Figure 3:**
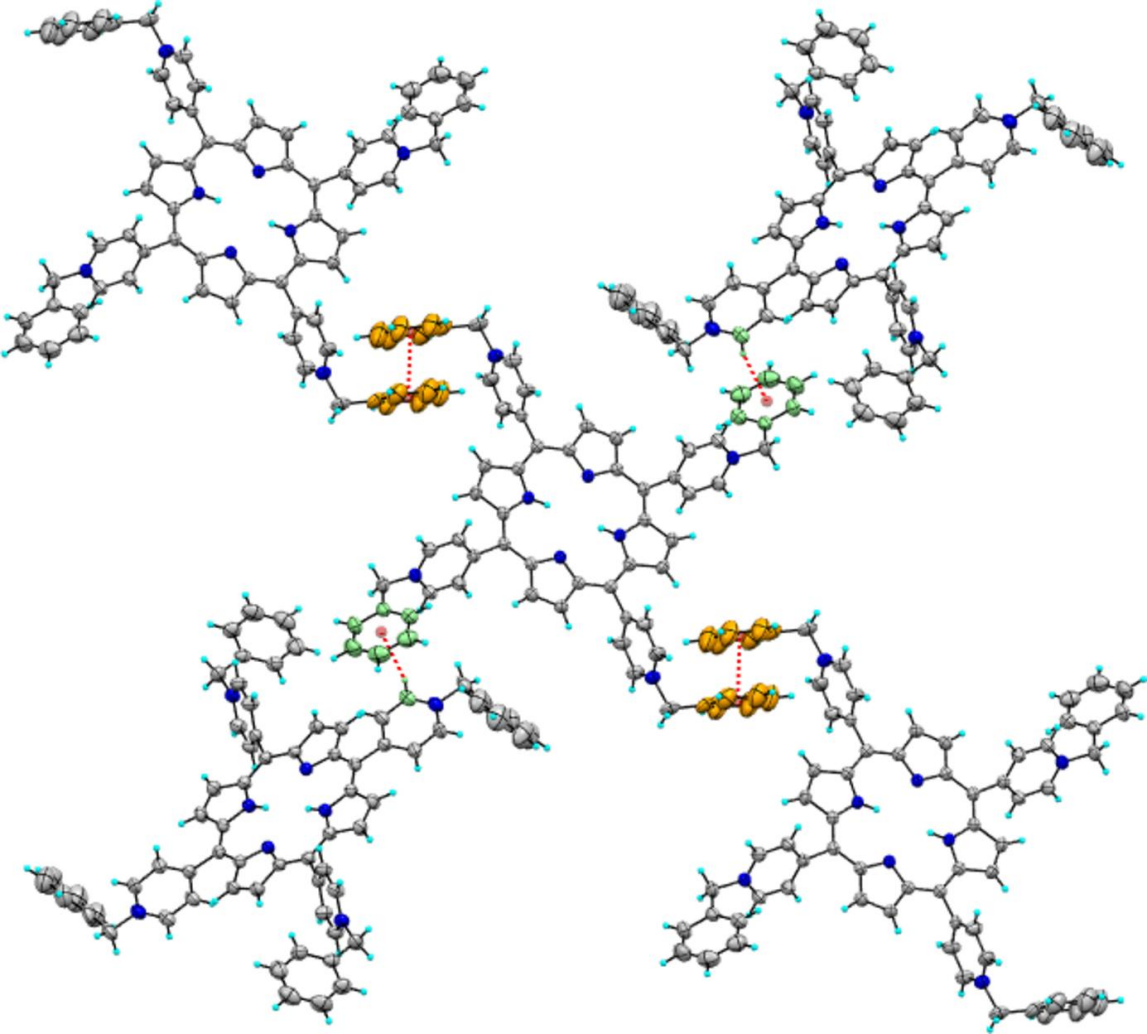
The inter­action of one porphyrin unit with four neighboring units through C—H⋯π contacts and π–π inter­actions.

The crystallographic results of **1**·OTf show that each porphyrin mol­ecule binds to four neighboring porphyrin units through C—H⋯π and π–π inter­actions. The N3-benzyl­pyridinium fragments are involved in C13—H13⋯π inter­ ;actions (Table 1 ▸) by means of the hydrogen atom of the pyridinium ring N3/C11–C15 adjacent to the positive charge (N^+^). According to the geometric parameters of C*sp* ^2^—H⋯π-systems, this inter­action is considered strong because the C13—H13⋯π distance is 2.65 Å and the C—H⋯π angle is 164°(Nishio, 2011 ▸; Nishio *et al.*, 2014 ▸; Brandl *et al.*, 2001 ▸). The π–π inter­action is through the stacking of the benzyl group bonded to the N4/C29–C34 pyridinium ring, where the centroid–centroid distance is 4.345 (4) Å. Fig. 3 ▸ shows the inter­action of a porphyrin unit with four neighboring units through C—H⋯π contacts and π–π inter­actions between the pyridinium and terminal phenyl groups.

**Figure 4:**
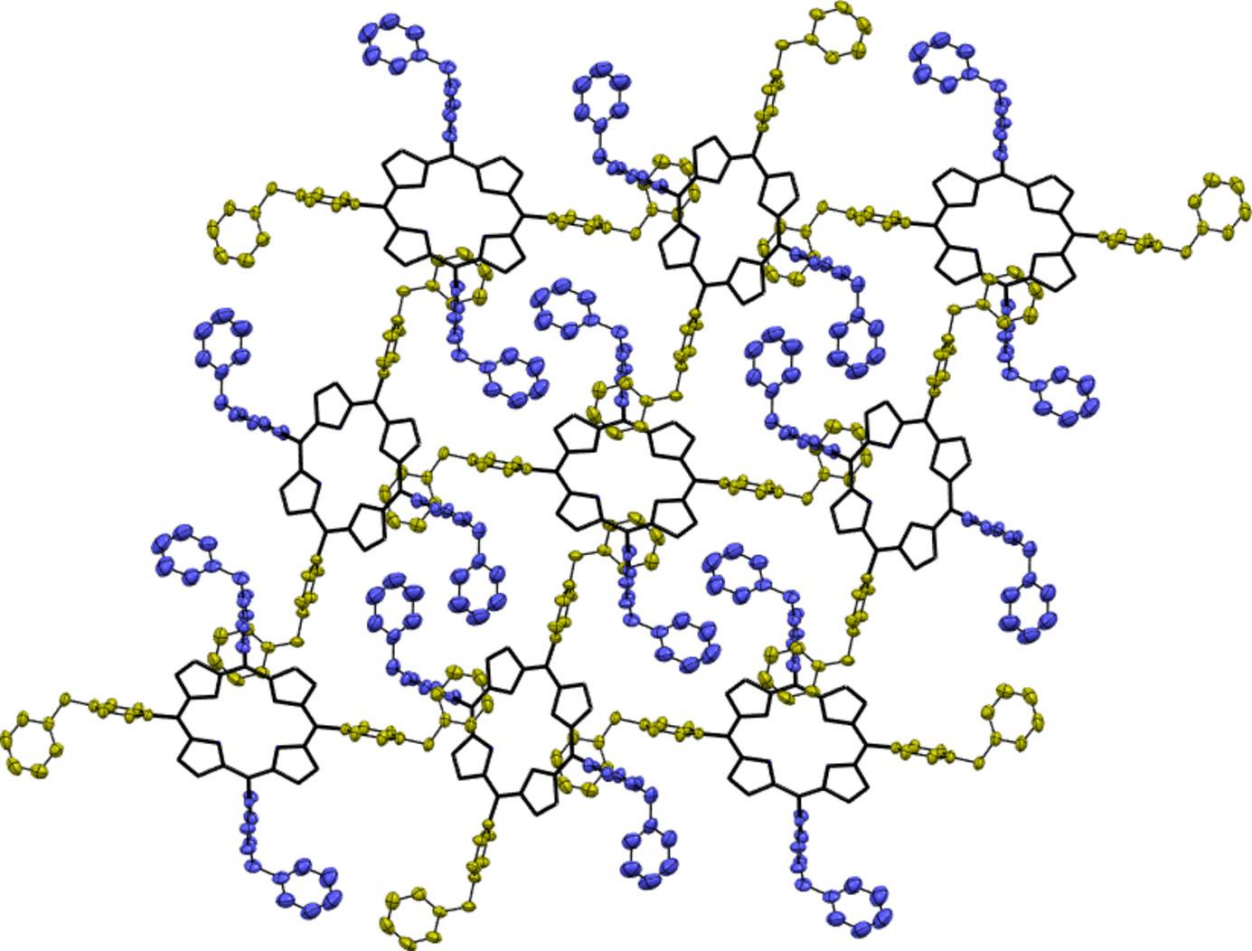
Square-grid two-dimensional lattice pattern observed in **1**·OTf. The N4-benzyl­pyridinium and N3-benzyl­pyridiniums groups are in green and blue, respectively. Hydrogen atoms have been omitted for clarity.

Those inter­actions in **1**·OTf lead to the formation of two-dimensional square-grid networks in which the squares are formed by N4-benzyl­pyridinium groups (green), while the N3-benzyl­pyridinium groups (blue) are placed inside each square and are separated from each other by 4.01 Å. This di-periodic lattice is illustrated in Fig. 4 ▸. Di-periodic square grid networks are common in free-base tetra­pyridyl­porphyrins (Lipstman & Goldberg, 2009*a* ▸, 2010 ▸).

**Figure 5:**
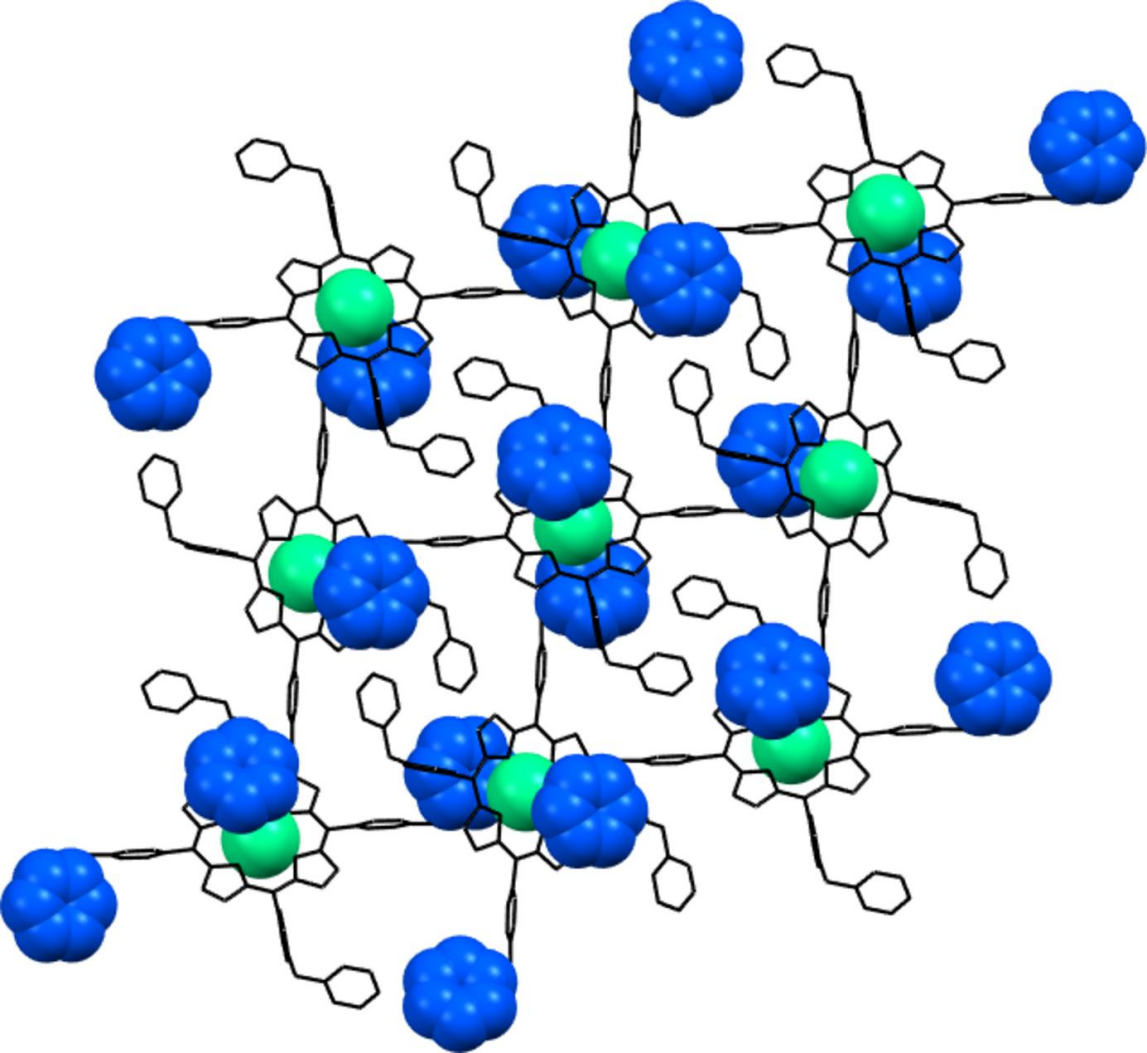
Ca^2+^⋯π contacts, shown in spacefilling mode, lead to di-periodic square-grid networks in **1**·OTf-Ca. Hydrogen atoms have been omitted for clarity.

In contrast to **1**·OTf-Ca, the phenyl group (C29–C34) bonded to the N4-pyridinium ring has a cation⋯π inter­action leading to di-periodic square-grid networks (Fig. 5 ▸). The Ca^2 +^⋯ π distance is 3.897  Å (distance between the cation and the centroid of the π-ring), θ ≤ 45° (the most preferred geometry is when the cation is on the π-system where θ = 0°) and α = 13.761° (α is the dihedral angle between the planes of the π-system and that of the cation), which are within the geometric parameters of the cation–π inter­action (Yamada, 2020 ▸; Borozan *et al.*, 2013 ▸). Fig. 4 ▸ illustrates the Ca^2+^ ⋯π inter­action in spacefilling mode.

## 4. Database survey

A search of the Cambridge Structural Database (version 5.44, April, 2024; Groom *et al.*, 2016 ▸) for related salts of 5,10,15,20-tetra (4-benzyl­pyridinium)-21*H*,23*H* porphyrin and its Ca^II^ complexes, revealed that no structures have been reported thus far (April 2024).

## 5. Synthesis and crystallization

A mixture of 5,10,15,20-tetra (4-pyrid­yl)-21*H*,23*H*-porphyrin (99.7 mg, 0.161 mmol) and 10.0 equiv. of benzyl bromide (280 mg, 1.61 mmol) in CH_3_CN (20.0 mL) was stirred under reflux for 24 h. Subsequently, 4.1 equiv. of silver triflate was added. The mixture reaction was filtered and the solvent was evaporated at r.t. for three days to give red–brown single crystals corresponding to **1**·Otf in a yield of 71%.

## 6. Refinement

Crystal data, data collection and structure refinement details are summarized in Table 2 ▸.The hydrogen atoms of the C—H and N—H bonds were placed in idealized positions whereas the hydrogen from water molecules were localized from the difference electron density map and their position was refined with *U*_iso_ tied to the parent atom with distance restraints (DFIX) *U*_iso_(H) = *aU*_eq_(parent atom) where *a* is 1.5 for –CH_3_ and N—H moieties and 1.2 for others.

## Supplementary Material

Crystal structure: contains datablock(s) I. DOI: 10.1107/S205698902400447X/ny2004sup1.cif

Structure factors: contains datablock(s) I. DOI: 10.1107/S205698902400447X/ny2004Isup2.hkl

CCDC reference: 2346689

Additional supporting information:  crystallographic information; 3D view; checkCIF report

## Figures and Tables

**Table 1:** Hydrogen-bond geometry (Å, °)

*D*—H⋯*A*	*D*—H	H⋯*A*	*D*⋯*A*	*D*—H⋯*A*
O7—H7*A*⋯O1	0.86 (2)	2.04 (2)	2.817 (5)	150 (3)
O7—H7*A*⋯O3*A*	0.86 (2)	2.14 (2)	2.874 (6)	143 (3)
O7—H7*B*⋯O4	0.88 (2)	2.24 (3)	2.965 (11)	140 (3)
O7—H7*B*⋯O6*A*	0.88 (2)	2.29 (3)	3.048 (10)	144 (3)
O8—H8*A*⋯O2^i^	0.87 (2)	2.08 (2)	2.914 (6)	163 (3)
O8—H8*A*⋯O2*A* ^i^	0.87 (2)	1.94 (2)	2.789 (7)	167 (3)
O8—H8*B*⋯O7	0.89 (2)	1.96 (2)	2.838 (3)	174 (3)

Symmetry code: (i) 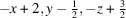 .

**Table 2:** Experimental details

Crystal data	
Chemical formula	[Ca_0.10_(C_68_H_53.81_N_8_)](CF_3_O_3_S)_4_·4H_2_O
*M*_r_	1655.20
Crystal system, space group	Monoclinic, *P*2_1_/*c*
Temperature (K)	293
*a*, *b*, *c* (Å)	10.4506 (4), 15.6166 (6), 22.3537 (9)
β (°)	90.417 (2)
*V* (Å^3^)	3648.1 (2)
*Z*	2
Radiation type	Cu *K*α
μ (mm^−1^)	2.18
Crystal size (mm)	0.49 × 0.29 × 0.15

Data collection	
Diffractometer	Bruker APEXII CCD
Absorption correction	Multi-scan (*SADABS*; Krause *et al.*, 2015 ▸)
*T*_min_, *T*_max_	0.586, 0.753
No. of measured, independent and observed [*I* > 2σ(*I*)] reflections	50051, 6684, 5995
*R*_int_	0.035
(sin θ/λ)_max_ (Å^−1^)	0.602

Refinement	
*R*[*F* ^2^ > 2σ(*F* ^2^)], *wR*(*F* ^2^), *S*	0.043, 0.126, 1.04
No. of reflections	6684
No. of parameters	791
No. of restraints	1623
H-atom treatment	H atoms treated by a mixture of independent and constrained refinement
Δρ_max_, Δρ_min_ (e Å^−3^)	0.22, −0.27

Computer programs: *APEX2* and *SAINT* (Bruker, 2019 ▸), *SHELXS97* and *SHELXTL* (Sheldrick 2008 ▸) and *SHELXL2019/2* (Sheldrick, 2015 ▸).

## supplementary crystallographic information

### Crystal data

**Table:**

[Ca_0.10_(C_68_H_53.81_N_8_)](CF_3_O_3_S)_4_·4H_2_O	*F*(000) = 1703
*M**_r_* = 1655.20	*D*_x_ = 1.507 Mg m^−^^3^
Monoclinic, *P*2_1_/*c*	Cu *K*α radiation, λ = 1.54178 Å
*a* = 10.4506 (4) Å	Cell parameters from 9998 reflections
*b* = 15.6166 (6) Å	θ = 3.5–69.4°
*c* = 22.3537 (9) Å	µ = 2.18 mm^−^^1^
β = 90.417 (2)°	*T* = 293 K
*V* = 3648.1 (2) Å^3^	Plate, red
*Z* = 2	0.49 × 0.28 × 0.15 mm

### Data collection

**Table:**

Bruker APEXII CCD diffractometer	6684 independent reflections
Radiation source: Incoatec ImuS	5995 reflections with *I* > 2σ(*I*)
Mirrors monochromator	*R*_int_ = 0.035
ω scans	θ_max_ = 68.2°, θ_min_ = 3.5°
Absorption correction: multi-scan (SADABS; Krause *et al.*, 2015)	*h* = −12→12
*T*_min_ = 0.586, *T*_max_ = 0.753	*k* = −18→18
50051 measured reflections	*l* = −26→26

### Refinement

**Table:**

Refinement on *F*^2^	Primary atom site location: structure-invariant direct methods
Least-squares matrix: full	Secondary atom site location: difference Fourier map
*R*[*F*^2^ > 2σ(*F*^2^)] = 0.043	Hydrogen site location: mixed
*wR*(*F*^2^) = 0.126	H atoms treated by a mixture of independent and constrained refinement
*S* = 1.03	*w* = 1/[σ^2^(*F*_o_^2^) + (0.0713*P*)^2^ + 1.1304*P*] where *P* = (*F*_o_^2^ + 2*F*_c_^2^)/3
6684 reflections	(Δ/σ)_max_ = 0.001
791 parameters	Δρ_max_ = 0.22 e Å^−^^3^
1623 restraints	Δρ_min_ = −0.27 e Å^−^^3^

### Special details

**Table:**

Geometry. All esds (except the esd in the dihedral angle between two l.s. planes) are estimated using the full covariance matrix. The cell esds are taken into account individually in the estimation of esds in distances, angles and torsion angles; correlations between esds in cell parameters are only used when they are defined by crystal symmetry. An approximate (isotropic) treatment of cell esds is used for estimating esds involving l.s. planes.

### Fractional atomic coordinates and isotropic or equivalent isotropic displacement parameters (Å^2^)

**Table:**

	*x*	*y*	*z*	*U*_iso_*/*U*_eq_	Occ. (<1)
Ca1	0.500000	1.000000	0.500000	0.0372 (17)	0.096 (2)
O7	0.80659 (19)	0.66609 (16)	0.72189 (10)	0.1002 (6)	
H7A	0.833 (3)	0.7028 (17)	0.6959 (12)	0.120*	
H7B	0.767 (3)	0.6257 (16)	0.7019 (14)	0.120*	
O8	1.04027 (18)	0.63868 (14)	0.78449 (9)	0.0916 (5)	
H8A	1.050 (3)	0.5840 (12)	0.7886 (15)	0.110*	
H8B	0.970 (2)	0.6459 (18)	0.7624 (13)	0.110*	
N1	0.59926 (11)	0.99267 (7)	0.42253 (5)	0.0354 (3)	
N2	0.56666 (12)	0.87932 (8)	0.52642 (6)	0.0368 (3)	
H2A	0.546 (2)	0.9301 (11)	0.5186 (10)	0.055*	0.904 (2)
N4	0.39344 (13)	0.69299 (8)	0.76441 (6)	0.0429 (3)	
C1	0.60390 (13)	1.05069 (9)	0.37688 (6)	0.0356 (3)	
C2	0.67922 (15)	1.01782 (10)	0.32765 (7)	0.0422 (3)	
H2	0.694744	1.045030	0.291413	0.051*	
C3	0.72193 (15)	0.94075 (10)	0.34459 (7)	0.0411 (3)	
H3	0.773680	0.904196	0.322554	0.049*	
C4	0.67194 (13)	0.92520 (9)	0.40395 (6)	0.0349 (3)	
C5	0.69278 (13)	0.84937 (9)	0.43609 (7)	0.0360 (3)	
C6	0.64139 (13)	0.82779 (9)	0.49145 (7)	0.0368 (3)	
C7	0.65604 (16)	0.74870 (10)	0.52261 (7)	0.0447 (4)	
H7	0.702550	0.701645	0.509449	0.054*	
C8	0.59099 (16)	0.75367 (10)	0.57446 (7)	0.0445 (4)	
H8	0.584306	0.710646	0.603031	0.053*	
C9	0.53414 (14)	0.83692 (9)	0.57764 (7)	0.0375 (3)	
C10	0.45622 (14)	0.86894 (9)	0.62318 (6)	0.0371 (3)	
C11	0.7804 (8)	0.7846 (4)	0.4085 (3)	0.0374 (12)	0.612 (10)
C12	0.9104 (9)	0.7940 (6)	0.4176 (3)	0.0481 (13)	0.612 (10)
H12	0.940570	0.838965	0.441047	0.058*	0.612 (10)
C13	0.9959 (8)	0.7378 (5)	0.3925 (3)	0.0504 (12)	0.612 (10)
H13	1.083126	0.745656	0.398884	0.060*	0.612 (10)
N3	0.9558 (7)	0.6728 (5)	0.3594 (3)	0.0508 (12)	0.612 (10)
C14	0.8305 (7)	0.6615 (5)	0.3485 (3)	0.0561 (14)	0.612 (10)
H14	0.803491	0.616189	0.324510	0.067*	0.612 (10)
C15	0.7418 (8)	0.7163 (6)	0.3726 (3)	0.0503 (14)	0.612 (10)
H15	0.655216	0.707711	0.364834	0.060*	0.612 (10)
C16	1.0498 (6)	0.6067 (4)	0.3377 (3)	0.0619 (12)	0.612 (10)
H16A	1.135832	0.629905	0.339932	0.074*	0.612 (10)
H16B	1.031262	0.592725	0.296310	0.074*	0.612 (10)
C17	1.0419 (4)	0.5266 (4)	0.3755 (3)	0.0632 (11)	0.612 (10)
C18	1.1090 (6)	0.5187 (4)	0.4289 (3)	0.0873 (15)	0.612 (10)
H18	1.160537	0.563880	0.441504	0.105*	0.612 (10)
C19	1.1021 (7)	0.4457 (4)	0.4644 (3)	0.1036 (17)	0.612 (10)
H19	1.147129	0.441612	0.500332	0.124*	0.612 (10)
C20	1.0256 (7)	0.3795 (5)	0.4440 (4)	0.1120 (17)	0.612 (10)
H20	1.018701	0.329820	0.466664	0.134*	0.612 (10)
C21	0.9596 (8)	0.3860 (5)	0.3909 (4)	0.1020 (17)	0.612 (10)
H21	0.910071	0.340266	0.377636	0.122*	0.612 (10)
C22	0.9659 (7)	0.4591 (5)	0.3571 (3)	0.0855 (16)	0.612 (10)
H22	0.918870	0.463208	0.321773	0.103*	0.612 (10)
C11A	0.7828 (12)	0.7861 (7)	0.4086 (6)	0.040 (2)	0.388 (10)
C12A	0.9133 (13)	0.7979 (9)	0.4049 (5)	0.0452 (17)	0.388 (10)
H12A	0.951268	0.847263	0.420082	0.054*	0.388 (10)
C13A	0.9868 (12)	0.7350 (8)	0.3780 (5)	0.0528 (19)	0.388 (10)
H13A	1.074842	0.742423	0.375102	0.063*	0.388 (10)
N3A	0.9330 (11)	0.6644 (7)	0.3563 (5)	0.0518 (17)	0.388 (10)
C14A	0.8065 (11)	0.6528 (8)	0.3594 (5)	0.0552 (18)	0.388 (10)
H14A	0.770732	0.603188	0.343482	0.066*	0.388 (10)
C15A	0.7288 (13)	0.7118 (9)	0.3855 (5)	0.0465 (17)	0.388 (10)
H15A	0.641053	0.702516	0.387672	0.056*	0.388 (10)
C16A	1.0104 (10)	0.5950 (7)	0.3270 (4)	0.0682 (19)	0.388 (10)
H16C	0.967560	0.576423	0.290598	0.082*	0.388 (10)
H16D	1.093687	0.617482	0.316204	0.082*	0.388 (10)
C17A	1.0272 (8)	0.5203 (7)	0.3681 (4)	0.0686 (18)	0.388 (10)
C18A	1.1372 (8)	0.5140 (5)	0.4025 (5)	0.0820 (18)	0.388 (10)
H18A	1.201016	0.555439	0.400538	0.098*	0.388 (10)
C19A	1.1489 (10)	0.4442 (6)	0.4397 (5)	0.101 (2)	0.388 (10)
H19A	1.222437	0.438679	0.463098	0.122*	0.388 (10)
C20A	1.0556 (11)	0.3822 (8)	0.4435 (5)	0.108 (2)	0.388 (10)
H20A	1.066684	0.335977	0.469277	0.129*	0.388 (10)
C21A	0.9457 (12)	0.3886 (8)	0.4092 (6)	0.101 (2)	0.388 (10)
H21A	0.881987	0.347167	0.411648	0.121*	0.388 (10)
C22A	0.9325 (10)	0.4580 (7)	0.3711 (5)	0.083 (2)	0.388 (10)
H22A	0.859548	0.462926	0.347272	0.099*	0.388 (10)
C23	0.43381 (14)	0.81063 (9)	0.67489 (7)	0.0381 (3)	
C24	0.53387 (15)	0.78163 (11)	0.71026 (7)	0.0440 (4)	
H24	0.616172	0.802527	0.704421	0.053*	
C25	0.51145 (16)	0.72196 (11)	0.75399 (7)	0.0467 (4)	
H25	0.579579	0.701332	0.776780	0.056*	
C26	0.29349 (16)	0.72276 (11)	0.73289 (8)	0.0495 (4)	
H26	0.211397	0.703608	0.741500	0.059*	
C27	0.31134 (15)	0.78131 (11)	0.68799 (8)	0.0474 (4)	
H27	0.241424	0.801500	0.666221	0.057*	
C28	0.37257 (19)	0.62615 (11)	0.81052 (8)	0.0508 (4)	
H28A	0.449629	0.591909	0.814403	0.061*	
H28B	0.304103	0.588619	0.797218	0.061*	
C29	0.33906 (15)	0.66198 (11)	0.87083 (7)	0.0434 (4)	
C30	0.28792 (19)	0.60547 (13)	0.91228 (8)	0.0565 (4)	
H30	0.273460	0.548720	0.901588	0.068*	
C31	0.2586 (2)	0.63286 (17)	0.96894 (9)	0.0712 (6)	
H31	0.223618	0.594628	0.996221	0.085*	
C32	0.2804 (2)	0.71607 (18)	0.98559 (9)	0.0730 (6)	
H32	0.260240	0.734238	1.024009	0.088*	
C33	0.3319 (2)	0.77247 (15)	0.94539 (10)	0.0674 (5)	
H33	0.347823	0.828791	0.956728	0.081*	
C34	0.36049 (17)	0.74569 (12)	0.88786 (9)	0.0536 (4)	
H34	0.394279	0.784401	0.860588	0.064*	
C35	0.9697 (7)	0.9025 (5)	0.5890 (3)	0.0690 (15)	0.567 (4)
F1	1.0514 (5)	0.9648 (4)	0.58440 (18)	0.1336 (18)	0.567 (4)
F2	1.0197 (6)	0.8344 (3)	0.5647 (2)	0.1305 (17)	0.567 (4)
F3	0.8743 (4)	0.9241 (3)	0.55352 (13)	0.1006 (10)	0.567 (4)
S1	0.9191 (2)	0.8838 (2)	0.66580 (13)	0.0600 (6)	0.567 (4)
O1	0.8216 (4)	0.8207 (3)	0.6574 (2)	0.1173 (15)	0.567 (4)
O2	0.8799 (6)	0.9651 (3)	0.6854 (2)	0.1189 (16)	0.567 (4)
O3	1.0294 (4)	0.8502 (4)	0.69258 (18)	0.1132 (15)	0.567 (4)
C35A	0.9759 (10)	0.9014 (7)	0.5917 (4)	0.075 (2)	0.433 (4)
F1A	1.0989 (3)	0.8891 (4)	0.59294 (18)	0.1000 (14)	0.433 (4)
F2A	0.9287 (7)	0.8438 (5)	0.5565 (2)	0.141 (2)	0.433 (4)
F3A	0.9587 (7)	0.9759 (4)	0.5681 (3)	0.134 (2)	0.433 (4)
S1A	0.9040 (4)	0.8957 (3)	0.6639 (2)	0.0908 (13)	0.433 (4)
O1A	0.7752 (4)	0.9047 (4)	0.6556 (2)	0.1127 (18)	0.433 (4)
O2A	0.9687 (7)	0.9624 (4)	0.6962 (3)	0.114 (2)	0.433 (4)
O3A	0.9478 (8)	0.8153 (4)	0.6862 (3)	0.124 (2)	0.433 (4)
C36	0.4698 (8)	0.5056 (5)	0.5924 (4)	0.0843 (17)	0.547 (12)
F4	0.4050 (9)	0.5732 (5)	0.5755 (4)	0.1231 (19)	0.547 (12)
F5	0.5709 (6)	0.5059 (6)	0.5597 (3)	0.121 (2)	0.547 (12)
F6	0.4130 (10)	0.4395 (4)	0.5706 (3)	0.121 (2)	0.547 (12)
S2	0.5069 (7)	0.4987 (3)	0.6715 (2)	0.0864 (13)	0.547 (12)
O4	0.5684 (12)	0.5790 (6)	0.6824 (6)	0.091 (2)	0.547 (12)
O5	0.5905 (11)	0.4265 (4)	0.6737 (3)	0.115 (2)	0.547 (12)
O6	0.3877 (9)	0.4856 (7)	0.6996 (4)	0.125 (3)	0.547 (12)
C36A	0.4403 (12)	0.5242 (7)	0.5903 (4)	0.090 (2)	0.453 (12)
F4A	0.3819 (10)	0.6009 (6)	0.5898 (5)	0.126 (3)	0.453 (12)
F5A	0.5411 (11)	0.5416 (6)	0.5586 (4)	0.124 (3)	0.453 (12)
F6A	0.3557 (10)	0.4696 (7)	0.5688 (4)	0.122 (3)	0.453 (12)
S2A	0.4801 (7)	0.5013 (3)	0.6668 (3)	0.0694 (9)	0.453 (12)
O4A	0.5144 (11)	0.4154 (4)	0.6694 (4)	0.114 (2)	0.453 (12)
O5A	0.3550 (8)	0.5159 (6)	0.6925 (4)	0.091 (2)	0.453 (12)
O6A	0.5726 (14)	0.5616 (9)	0.6850 (8)	0.098 (3)	0.453 (12)

### Atomic displacement parameters (Å^2^)

**Table:**

	*U*^11^	*U*^22^	*U*^33^	*U*^12^	*U*^13^	*U*^23^
Ca1	0.044 (3)	0.030 (2)	0.037 (3)	0.0098 (16)	0.0126 (17)	0.0093 (17)
O7	0.0761 (11)	0.1164 (17)	0.1084 (15)	0.0033 (10)	0.0128 (10)	0.0417 (13)
O8	0.0759 (11)	0.1117 (14)	0.0873 (12)	−0.0215 (11)	0.0056 (9)	0.0076 (12)
N1	0.0385 (6)	0.0301 (6)	0.0376 (6)	0.0035 (5)	0.0065 (5)	0.0050 (5)
N2	0.0401 (6)	0.0306 (6)	0.0399 (6)	0.0069 (5)	0.0082 (5)	0.0080 (5)
N4	0.0508 (7)	0.0382 (7)	0.0399 (7)	0.0066 (5)	0.0117 (6)	0.0104 (5)
C1	0.0364 (7)	0.0339 (7)	0.0366 (7)	0.0006 (5)	0.0052 (6)	0.0055 (6)
C2	0.0504 (8)	0.0398 (8)	0.0365 (8)	0.0024 (6)	0.0100 (6)	0.0056 (6)
C3	0.0458 (8)	0.0377 (8)	0.0401 (8)	0.0044 (6)	0.0118 (6)	0.0007 (6)
C4	0.0349 (7)	0.0326 (7)	0.0373 (7)	0.0012 (5)	0.0053 (5)	0.0020 (6)
C5	0.0356 (7)	0.0317 (7)	0.0408 (8)	0.0030 (6)	0.0043 (6)	0.0011 (6)
C6	0.0371 (7)	0.0326 (7)	0.0407 (8)	0.0048 (5)	0.0048 (6)	0.0041 (6)
C7	0.0521 (9)	0.0337 (7)	0.0484 (9)	0.0130 (6)	0.0090 (7)	0.0060 (7)
C8	0.0545 (9)	0.0334 (7)	0.0456 (8)	0.0086 (6)	0.0080 (7)	0.0113 (6)
C9	0.0397 (7)	0.0336 (7)	0.0394 (8)	0.0034 (6)	0.0052 (6)	0.0080 (6)
C10	0.0386 (7)	0.0346 (7)	0.0381 (7)	0.0018 (6)	0.0045 (6)	0.0081 (6)
C11	0.042 (2)	0.032 (2)	0.038 (2)	0.007 (2)	0.007 (2)	0.009 (2)
C12	0.0457 (19)	0.054 (2)	0.044 (3)	0.0071 (17)	0.007 (2)	−0.007 (2)
C13	0.0452 (18)	0.062 (2)	0.044 (3)	0.0094 (15)	0.0027 (18)	−0.004 (2)
N3	0.050 (2)	0.046 (2)	0.0563 (19)	0.0123 (17)	0.0144 (16)	0.0042 (16)
C14	0.066 (3)	0.041 (2)	0.061 (3)	0.000 (2)	0.022 (2)	−0.013 (2)
C15	0.047 (2)	0.047 (2)	0.058 (3)	−0.0005 (17)	0.008 (2)	−0.009 (2)
C16	0.057 (2)	0.061 (2)	0.068 (2)	0.0204 (19)	0.0158 (19)	−0.0117 (18)
C17	0.062 (2)	0.048 (2)	0.080 (2)	0.0226 (18)	0.0039 (19)	−0.0120 (18)
C18	0.086 (3)	0.070 (2)	0.105 (3)	0.005 (2)	−0.022 (3)	0.006 (3)
C19	0.107 (4)	0.085 (3)	0.118 (4)	0.021 (3)	−0.030 (3)	0.019 (3)
C20	0.115 (4)	0.068 (3)	0.153 (4)	0.013 (3)	−0.020 (3)	0.025 (3)
C21	0.115 (3)	0.063 (2)	0.128 (4)	−0.002 (2)	−0.014 (3)	−0.012 (3)
C22	0.098 (4)	0.068 (2)	0.090 (3)	0.011 (3)	−0.007 (3)	−0.019 (2)
C11A	0.045 (3)	0.036 (4)	0.040 (4)	0.007 (3)	0.007 (4)	0.003 (3)
C12A	0.042 (3)	0.048 (3)	0.046 (4)	0.002 (2)	0.010 (3)	0.003 (3)
C13A	0.049 (3)	0.063 (3)	0.047 (4)	0.016 (3)	0.014 (3)	0.005 (3)
N3A	0.056 (3)	0.045 (3)	0.055 (3)	0.015 (3)	0.024 (3)	0.005 (2)
C14A	0.062 (3)	0.041 (3)	0.063 (4)	0.001 (3)	0.027 (3)	0.001 (3)
C15A	0.050 (3)	0.038 (3)	0.051 (4)	0.005 (2)	0.021 (3)	−0.003 (3)
C16A	0.070 (4)	0.065 (3)	0.070 (3)	0.024 (3)	0.018 (3)	−0.005 (3)
C17A	0.073 (3)	0.052 (3)	0.081 (3)	0.022 (3)	0.001 (3)	−0.014 (3)
C18A	0.076 (3)	0.058 (3)	0.112 (4)	0.018 (3)	−0.010 (3)	0.009 (3)
C19A	0.090 (4)	0.073 (3)	0.141 (5)	0.021 (3)	−0.027 (4)	0.025 (4)
C20A	0.102 (4)	0.067 (4)	0.154 (4)	0.017 (3)	−0.024 (4)	0.026 (4)
C21A	0.106 (4)	0.068 (3)	0.129 (5)	0.004 (3)	−0.013 (4)	0.011 (4)
C22A	0.082 (4)	0.067 (3)	0.099 (4)	0.014 (3)	−0.005 (3)	−0.005 (3)
C23	0.0429 (8)	0.0335 (7)	0.0379 (7)	0.0049 (6)	0.0083 (6)	0.0054 (6)
C24	0.0396 (8)	0.0484 (9)	0.0440 (8)	0.0024 (6)	0.0072 (6)	0.0107 (7)
C25	0.0457 (8)	0.0521 (9)	0.0424 (8)	0.0100 (7)	0.0046 (6)	0.0121 (7)
C26	0.0425 (8)	0.0504 (9)	0.0558 (10)	−0.0014 (7)	0.0105 (7)	0.0133 (8)
C27	0.0404 (8)	0.0492 (9)	0.0526 (9)	0.0041 (7)	0.0028 (7)	0.0153 (7)
C28	0.0670 (11)	0.0384 (8)	0.0472 (9)	0.0053 (7)	0.0139 (8)	0.0137 (7)
C29	0.0401 (8)	0.0475 (9)	0.0428 (8)	0.0024 (6)	0.0042 (6)	0.0106 (7)
C30	0.0639 (11)	0.0555 (10)	0.0503 (10)	−0.0009 (8)	0.0088 (8)	0.0174 (8)
C31	0.0764 (13)	0.0886 (16)	0.0488 (11)	0.0090 (12)	0.0159 (9)	0.0250 (11)
C32	0.0722 (13)	0.1041 (18)	0.0428 (10)	0.0168 (12)	0.0035 (9)	−0.0010 (11)
C33	0.0629 (12)	0.0715 (13)	0.0677 (13)	0.0035 (10)	−0.0010 (10)	−0.0145 (11)
C34	0.0506 (9)	0.0519 (10)	0.0584 (10)	−0.0035 (7)	0.0103 (8)	0.0039 (8)
C35	0.064 (3)	0.077 (3)	0.066 (3)	−0.005 (3)	0.014 (2)	−0.009 (3)
F1	0.136 (3)	0.155 (4)	0.109 (3)	−0.087 (3)	0.011 (3)	0.012 (3)
F2	0.158 (4)	0.116 (3)	0.118 (3)	0.044 (3)	0.059 (3)	−0.018 (2)
F3	0.116 (2)	0.119 (3)	0.0660 (15)	0.000 (2)	−0.0130 (15)	−0.0059 (16)
S1	0.0418 (6)	0.0827 (13)	0.0557 (10)	0.0037 (7)	0.0140 (5)	0.0009 (8)
O1	0.094 (3)	0.140 (4)	0.118 (3)	−0.049 (3)	0.016 (2)	0.024 (3)
O2	0.151 (4)	0.108 (3)	0.098 (3)	0.044 (3)	0.017 (3)	−0.036 (2)
O3	0.079 (2)	0.174 (4)	0.086 (2)	0.033 (3)	−0.0029 (19)	0.013 (3)
C35A	0.070 (3)	0.084 (4)	0.070 (4)	−0.002 (3)	−0.004 (3)	−0.003 (3)
F1A	0.0652 (19)	0.153 (4)	0.082 (2)	0.019 (2)	0.0136 (16)	−0.003 (3)
F2A	0.137 (4)	0.190 (5)	0.096 (3)	−0.030 (4)	−0.010 (3)	−0.069 (3)
F3A	0.141 (5)	0.136 (4)	0.126 (4)	0.039 (4)	0.049 (4)	0.069 (3)
S1A	0.109 (3)	0.087 (2)	0.0757 (17)	−0.0344 (18)	−0.0129 (16)	0.0161 (14)
O1A	0.073 (3)	0.150 (5)	0.116 (4)	−0.024 (3)	0.033 (2)	0.007 (3)
O2A	0.128 (4)	0.126 (4)	0.088 (3)	−0.041 (4)	0.019 (3)	−0.029 (3)
O3A	0.162 (6)	0.093 (3)	0.117 (4)	−0.014 (4)	−0.003 (4)	0.048 (3)
C36	0.110 (4)	0.067 (3)	0.076 (3)	−0.003 (3)	0.001 (3)	0.004 (2)
F4	0.160 (4)	0.092 (4)	0.117 (4)	0.034 (3)	−0.041 (3)	−0.009 (3)
F5	0.124 (3)	0.169 (6)	0.071 (2)	0.025 (3)	0.0232 (19)	0.011 (4)
F6	0.194 (6)	0.093 (3)	0.075 (2)	−0.041 (3)	−0.013 (4)	−0.009 (2)
S2	0.137 (3)	0.0700 (16)	0.0527 (11)	0.0024 (14)	0.0088 (16)	−0.0095 (9)
O4	0.129 (4)	0.053 (3)	0.091 (3)	0.010 (2)	−0.003 (3)	−0.010 (2)
O5	0.191 (6)	0.062 (2)	0.091 (3)	0.031 (3)	−0.018 (4)	−0.010 (2)
O6	0.142 (5)	0.155 (6)	0.080 (3)	−0.033 (4)	0.026 (4)	0.007 (4)
C36A	0.131 (4)	0.072 (4)	0.068 (3)	−0.026 (4)	−0.007 (3)	0.017 (3)
F4A	0.136 (5)	0.099 (5)	0.144 (6)	−0.015 (4)	−0.027 (4)	0.026 (4)
F5A	0.169 (6)	0.117 (5)	0.085 (3)	−0.039 (4)	0.036 (4)	−0.001 (4)
F6A	0.156 (6)	0.132 (5)	0.078 (3)	−0.060 (4)	−0.017 (4)	−0.001 (4)
S2A	0.1048 (19)	0.0401 (12)	0.0632 (16)	−0.0050 (10)	−0.0020 (11)	−0.0086 (9)
O4A	0.162 (6)	0.056 (3)	0.125 (4)	0.018 (4)	−0.023 (5)	−0.012 (2)
O5A	0.111 (4)	0.094 (4)	0.069 (3)	0.005 (3)	0.010 (3)	−0.021 (3)
O6A	0.111 (5)	0.071 (6)	0.111 (5)	−0.008 (4)	−0.037 (4)	−0.019 (5)

### Geometric parameters (Å, º)

**Table:**

Ca1—N1^i^	2.0283 (12)	C13A—N3A	1.329 (11)
Ca1—N1	2.0284 (12)	C13A—H13A	0.9300
Ca1—N2^i^	2.0927 (12)	N3A—C14A	1.337 (10)
Ca1—N2	2.0928 (12)	N3A—C16A	1.505 (10)
Ca1—C4	3.0430 (14)	C14A—C15A	1.362 (11)
Ca1—C4^i^	3.0430 (14)	C14A—H14A	0.9300
Ca1—C1	3.0698 (14)	C15A—H15A	0.9300
Ca1—C1^i^	3.0698 (14)	C16A—C17A	1.495 (10)
Ca1—C6	3.0751 (14)	C16A—H16C	0.9700
Ca1—C6^i^	3.0751 (14)	C16A—H16D	0.9700
Ca1—C9^i^	3.1010 (14)	C17A—C18A	1.382 (7)
Ca1—C9	3.1011 (14)	C17A—C22A	1.390 (7)
Ca1—H2A	1.263 (17)	C18A—C19A	1.377 (7)
Ca1—H2A^i^	1.263 (17)	C18A—H18A	0.9300
O7—H7A	0.862 (18)	C19A—C20A	1.377 (8)
O7—H7B	0.875 (17)	C19A—H19A	0.9300
O8—H8A	0.865 (17)	C20A—C21A	1.380 (8)
O8—H8B	0.885 (17)	C20A—H20A	0.9300
N1—C4	1.3653 (18)	C21A—C22A	1.385 (7)
N1—C1	1.3658 (18)	C21A—H21A	0.9300
N2—C9	1.3677 (19)	C22A—H22A	0.9300
N2—C6	1.3702 (19)	C23—C24	1.383 (2)
N2—H2A	0.839 (16)	C23—C27	1.392 (2)
N4—C25	1.336 (2)	C24—C25	1.372 (2)
N4—C26	1.339 (2)	C24—H24	0.9300
N4—C28	1.4843 (19)	C25—H25	0.9300
C1—C10^i^	1.404 (2)	C26—C27	1.371 (2)
C1—C2	1.451 (2)	C26—H26	0.9300
C2—C3	1.337 (2)	C27—H27	0.9300
C2—H2	0.9300	C28—C29	1.503 (2)
C3—C4	1.450 (2)	C28—H28A	0.9700
C3—H3	0.9300	C28—H28B	0.9700
C4—C5	1.401 (2)	C29—C34	1.379 (3)
C5—C6	1.394 (2)	C29—C30	1.389 (2)
C5—C11A	1.499 (2)	C30—C31	1.374 (3)
C5—C11	1.500 (2)	C30—H30	0.9300
C6—C7	1.426 (2)	C31—C32	1.370 (4)
C7—C8	1.350 (2)	C31—H31	0.9300
C7—H7	0.9300	C32—C33	1.372 (3)
C8—C9	1.431 (2)	C32—H32	0.9300
C8—H8	0.9300	C33—C34	1.387 (3)
C9—C10	1.401 (2)	C33—H33	0.9300
C10—C23	1.491 (2)	C34—H34	0.9300
C11—C12	1.380 (7)	C35—F1	1.298 (8)
C11—C15	1.393 (7)	C35—F2	1.305 (8)
C12—C13	1.375 (7)	C35—F3	1.314 (8)
C12—H12	0.9300	C35—S1	1.823 (7)
C13—N3	1.323 (7)	S1—O3	1.396 (4)
C13—H13	0.9300	S1—O2	1.405 (5)
N3—C14	1.342 (7)	S1—O1	1.429 (5)
N3—C16	1.506 (6)	C35A—F3A	1.289 (10)
C14—C15	1.375 (7)	C35A—F2A	1.292 (11)
C14—H14	0.9300	C35A—F1A	1.299 (11)
C15—H15	0.9300	C35A—S1A	1.786 (10)
C16—C17	1.513 (7)	S1A—O1A	1.365 (6)
C16—H16A	0.9700	S1A—O3A	1.425 (7)
C16—H16B	0.9700	S1A—O2A	1.434 (7)
C17—C22	1.381 (6)	C36—F6	1.285 (8)
C17—C18	1.385 (6)	C36—F5	1.288 (8)
C18—C19	1.391 (6)	C36—F4	1.309 (8)
C18—H18	0.9300	C36—S2	1.812 (8)
C19—C20	1.382 (6)	S2—O6	1.414 (8)
C19—H19	0.9300	S2—O5	1.426 (7)
C20—C21	1.373 (6)	S2—O4	1.430 (8)
C20—H20	0.9300	C36A—F5A	1.302 (10)
C21—C22	1.369 (6)	C36A—F6A	1.318 (9)
C21—H21	0.9300	C36A—F4A	1.345 (10)
C22—H22	0.9300	C36A—S2A	1.793 (9)
C11A—C12A	1.379 (11)	S2A—O4A	1.389 (8)
C11A—C15A	1.388 (12)	S2A—O6A	1.408 (9)
C12A—C13A	1.387 (11)	S2A—O5A	1.449 (8)
C12A—H12A	0.9300		
			
N1^i^—Ca1—N1	180.0	C14—N3—C16	119.3 (6)
N1^i^—Ca1—N2^i^	91.08 (5)	N3—C14—C15	120.5 (6)
N1—Ca1—N2^i^	88.92 (5)	N3—C14—H14	119.8
N1^i^—Ca1—N2	88.92 (5)	C15—C14—H14	119.8
N1—Ca1—N2	91.08 (5)	C14—C15—C11	120.7 (6)
N2^i^—Ca1—N2	180.00 (7)	C14—C15—H15	119.7
N1^i^—Ca1—C4	158.81 (4)	C11—C15—H15	119.7
N1—Ca1—C4	21.19 (4)	N3—C16—C17	110.4 (4)
N2^i^—Ca1—C4	110.10 (4)	N3—C16—H16A	109.6
N2—Ca1—C4	69.90 (4)	C17—C16—H16A	109.6
N1^i^—Ca1—C4^i^	21.19 (4)	N3—C16—H16B	109.6
N1—Ca1—C4^i^	158.81 (4)	C17—C16—H16B	109.6
N2^i^—Ca1—C4^i^	69.90 (4)	H16A—C16—H16B	108.1
N2—Ca1—C4^i^	110.10 (4)	C22—C17—C18	118.4 (6)
C4—Ca1—C4^i^	180.0	C22—C17—C16	120.0 (5)
N1^i^—Ca1—C1	159.60 (4)	C18—C17—C16	121.7 (5)
N1—Ca1—C1	20.40 (4)	C17—C18—C19	122.4 (5)
N2^i^—Ca1—C1	68.53 (4)	C17—C18—H18	118.8
N2—Ca1—C1	111.47 (4)	C19—C18—H18	118.8
C4—Ca1—C1	41.57 (4)	C20—C19—C18	117.2 (6)
C4^i^—Ca1—C1	138.43 (4)	C20—C19—H19	121.4
N1^i^—Ca1—C1^i^	20.40 (4)	C18—C19—H19	121.4
N1—Ca1—C1^i^	159.60 (4)	C21—C20—C19	121.0 (8)
N2^i^—Ca1—C1^i^	111.47 (4)	C21—C20—H20	119.5
N2—Ca1—C1^i^	68.53 (4)	C19—C20—H20	119.5
C4—Ca1—C1^i^	138.43 (4)	C22—C21—C20	120.8 (7)
C4^i^—Ca1—C1^i^	41.57 (4)	C22—C21—H21	119.6
C1—Ca1—C1^i^	180.0	C20—C21—H21	119.6
N1^i^—Ca1—C6	110.57 (4)	C21—C22—C17	120.1 (6)
N1—Ca1—C6	69.43 (4)	C21—C22—H22	119.9
N2^i^—Ca1—C6	158.30 (4)	C17—C22—H22	119.9
N2—Ca1—C6	21.70 (4)	C12A—C11A—C15A	119.3 (7)
C4—Ca1—C6	48.24 (4)	C12A—C11A—C5	124.0 (11)
C4^i^—Ca1—C6	131.76 (4)	C15A—C11A—C5	116.7 (11)
C1—Ca1—C6	89.80 (4)	C11A—C12A—C13A	118.8 (10)
C1^i^—Ca1—C6	90.20 (4)	C11A—C12A—H12A	120.6
N1^i^—Ca1—C6^i^	69.43 (4)	C13A—C12A—H12A	120.6
N1—Ca1—C6^i^	110.57 (4)	N3A—C13A—C12A	120.8 (10)
N2^i^—Ca1—C6^i^	21.70 (4)	N3A—C13A—H13A	119.6
N2—Ca1—C6^i^	158.30 (4)	C12A—C13A—H13A	119.6
C4—Ca1—C6^i^	131.76 (4)	C13A—N3A—C14A	120.6 (9)
C4^i^—Ca1—C6^i^	48.24 (4)	C13A—N3A—C16A	122.0 (10)
C1—Ca1—C6^i^	90.20 (4)	C14A—N3A—C16A	117.4 (9)
C1^i^—Ca1—C6^i^	89.80 (4)	N3A—C14A—C15A	121.6 (10)
C6—Ca1—C6^i^	180.0	N3A—C14A—H14A	119.2
N1^i^—Ca1—C9^i^	111.96 (4)	C15A—C14A—H14A	119.2
N1—Ca1—C9^i^	68.04 (4)	C14A—C15A—C11A	118.8 (10)
N2^i^—Ca1—C9^i^	20.90 (4)	C14A—C15A—H15A	120.6
N2—Ca1—C9^i^	159.10 (4)	C11A—C15A—H15A	120.6
C4—Ca1—C9^i^	89.23 (4)	C17A—C16A—N3A	110.8 (6)
C4^i^—Ca1—C9^i^	90.77 (4)	C17A—C16A—H16C	109.5
C1—Ca1—C9^i^	47.66 (4)	N3A—C16A—H16C	109.5
C1^i^—Ca1—C9^i^	132.34 (4)	C17A—C16A—H16D	109.5
C6—Ca1—C9^i^	137.46 (4)	N3A—C16A—H16D	109.5
C6^i^—Ca1—C9^i^	42.54 (4)	H16C—C16A—H16D	108.1
N1^i^—Ca1—C9	68.04 (4)	C18A—C17A—C22A	120.9 (9)
N1—Ca1—C9	111.96 (4)	C18A—C17A—C16A	119.4 (8)
N2^i^—Ca1—C9	159.10 (4)	C22A—C17A—C16A	119.7 (8)
N2—Ca1—C9	20.90 (4)	C19A—C18A—C17A	117.5 (8)
C4—Ca1—C9	90.77 (4)	C19A—C18A—H18A	121.2
C4^i^—Ca1—C9	89.23 (4)	C17A—C18A—H18A	121.2
C1—Ca1—C9	132.34 (4)	C18A—C19A—C20A	122.3 (9)
C1^i^—Ca1—C9	47.66 (4)	C18A—C19A—H19A	118.8
C6—Ca1—C9	42.54 (4)	C20A—C19A—H19A	118.8
C6^i^—Ca1—C9	137.46 (4)	C19A—C20A—C21A	120.1 (11)
C9^i^—Ca1—C9	180.0	C19A—C20A—H20A	119.9
N1^i^—Ca1—H2A	87.9 (10)	C21A—C20A—H20A	119.9
N1—Ca1—H2A	92.1 (10)	C20A—C21A—C22A	118.5 (11)
N2^i^—Ca1—H2A	175.7 (10)	C20A—C21A—H21A	120.8
N2—Ca1—H2A	4.3 (10)	C22A—C21A—H21A	120.8
C4—Ca1—H2A	71.0 (10)	C21A—C22A—C17A	120.6 (10)
C4^i^—Ca1—H2A	109.0 (10)	C21A—C22A—H22A	119.7
C1—Ca1—H2A	112.4 (10)	C17A—C22A—H22A	119.7
C1^i^—Ca1—H2A	67.6 (10)	C24—C23—C27	117.68 (14)
C6—Ca1—H2A	23.3 (10)	C24—C23—C10	121.39 (13)
C6^i^—Ca1—H2A	156.7 (10)	C27—C23—C10	120.90 (14)
C9^i^—Ca1—H2A	159.5 (10)	C25—C24—C23	119.84 (15)
C9—Ca1—H2A	20.5 (10)	C25—C24—H24	120.1
N1^i^—Ca1—H2A^i^	92.1 (10)	C23—C24—H24	120.1
N1—Ca1—H2A^i^	87.9 (10)	N4—C25—C24	121.13 (15)
N2^i^—Ca1—H2A^i^	4.3 (10)	N4—C25—H25	119.4
N2—Ca1—H2A^i^	175.7 (10)	C24—C25—H25	119.4
C4—Ca1—H2A^i^	109.0 (10)	N4—C26—C27	120.45 (15)
C4^i^—Ca1—H2A^i^	71.0 (10)	N4—C26—H26	119.8
C1—Ca1—H2A^i^	67.6 (10)	C27—C26—H26	119.8
C1^i^—Ca1—H2A^i^	112.4 (10)	C26—C27—C23	120.22 (15)
C6—Ca1—H2A^i^	156.7 (10)	C26—C27—H27	119.9
C6^i^—Ca1—H2A^i^	23.3 (10)	C23—C27—H27	119.9
C9^i^—Ca1—H2A^i^	20.5 (10)	N4—C28—C29	113.42 (14)
C9—Ca1—H2A^i^	159.5 (10)	N4—C28—H28A	108.9
H2A—Ca1—H2A^i^	179.997 (11)	C29—C28—H28A	108.9
H7A—O7—H7B	107 (2)	N4—C28—H28B	108.9
H8A—O8—H8B	106 (2)	C29—C28—H28B	108.9
C4—N1—C1	105.18 (12)	H28A—C28—H28B	107.7
C4—N1—Ca1	126.33 (9)	C34—C29—C30	118.72 (17)
C1—N1—Ca1	128.43 (9)	C34—C29—C28	124.18 (15)
C9—N2—C6	109.83 (12)	C30—C29—C28	117.05 (16)
C9—N2—Ca1	126.02 (10)	C31—C30—C29	120.4 (2)
C6—N2—Ca1	123.91 (10)	C31—C30—H30	119.8
C9—N2—H2A	124.5 (16)	C29—C30—H30	119.8
C6—N2—H2A	125.6 (16)	C32—C31—C30	120.5 (2)
Ca1—N2—H2A	6.5 (16)	C32—C31—H31	119.7
C25—N4—C26	120.56 (13)	C30—C31—H31	119.7
C25—N4—C28	119.98 (14)	C31—C32—C33	119.8 (2)
C26—N4—C28	119.45 (14)	C31—C32—H32	120.1
N1—C1—C10^i^	125.15 (13)	C33—C32—H32	120.1
N1—C1—C2	110.75 (12)	C32—C33—C34	120.1 (2)
C10^i^—C1—C2	124.10 (13)	C32—C33—H33	119.9
N1—C1—Ca1	31.17 (6)	C34—C33—H33	119.9
C10^i^—C1—Ca1	94.03 (9)	C29—C34—C33	120.40 (18)
C2—C1—Ca1	141.80 (10)	C29—C34—H34	119.8
C3—C2—C1	106.57 (13)	C33—C34—H34	119.8
C3—C2—H2	126.7	F1—C35—F2	108.2 (6)
C1—C2—H2	126.7	F1—C35—F3	104.9 (6)
C2—C3—C4	106.74 (13)	F2—C35—F3	105.2 (6)
C2—C3—H3	126.6	F1—C35—S1	113.0 (5)
C4—C3—H3	126.6	F2—C35—S1	112.3 (5)
N1—C4—C5	125.51 (13)	F3—C35—S1	112.7 (5)
N1—C4—C3	110.74 (12)	O3—S1—O2	116.5 (4)
C5—C4—C3	123.71 (13)	O3—S1—O1	112.6 (4)
N1—C4—Ca1	32.48 (6)	O2—S1—O1	117.0 (4)
C5—C4—Ca1	93.04 (9)	O3—S1—C35	102.7 (3)
C3—C4—Ca1	143.12 (10)	O2—S1—C35	103.6 (4)
C6—C5—C4	126.87 (13)	O1—S1—C35	101.5 (3)
C6—C5—C11A	116.8 (5)	F3A—C35A—F2A	109.1 (9)
C4—C5—C11A	116.3 (5)	F3A—C35A—F1A	106.1 (8)
C6—C5—C11	116.3 (3)	F2A—C35A—F1A	106.4 (8)
C4—C5—C11	116.9 (3)	F3A—C35A—S1A	110.9 (7)
N2—C6—C5	126.11 (13)	F2A—C35A—S1A	110.8 (7)
N2—C6—C7	106.85 (12)	F1A—C35A—S1A	113.3 (7)
C5—C6—C7	127.05 (13)	O1A—S1A—O3A	116.9 (5)
N2—C6—Ca1	34.39 (6)	O1A—S1A—O2A	117.0 (6)
C5—C6—Ca1	91.84 (9)	O3A—S1A—O2A	108.3 (5)
C7—C6—Ca1	141.01 (10)	O1A—S1A—C35A	107.0 (5)
C8—C7—C6	108.46 (13)	O3A—S1A—C35A	103.0 (5)
C8—C7—H7	125.8	O2A—S1A—C35A	102.6 (5)
C6—C7—H7	125.8	F6—C36—F5	99.7 (8)
C7—C8—C9	107.86 (13)	F6—C36—F4	107.6 (7)
C7—C8—H8	126.1	F5—C36—F4	105.0 (7)
C9—C8—H8	126.1	F6—C36—S2	114.6 (6)
N2—C9—C10	125.80 (13)	F5—C36—S2	112.5 (6)
N2—C9—C8	107.00 (13)	F4—C36—S2	115.9 (6)
C10—C9—C8	127.17 (14)	O6—S2—O5	114.3 (6)
N2—C9—Ca1	33.08 (6)	O6—S2—O4	116.6 (7)
C10—C9—Ca1	92.77 (9)	O5—S2—O4	114.4 (7)
C8—C9—Ca1	139.84 (10)	O6—S2—C36	105.0 (6)
C9—C10—C1^i^	125.54 (13)	O5—S2—C36	102.0 (4)
C9—C10—C23	116.15 (13)	O4—S2—C36	101.9 (7)
C1^i^—C10—C23	118.29 (13)	F5A—C36A—F6A	118.7 (9)
C12—C11—C15	116.5 (5)	F5A—C36A—F4A	100.2 (8)
C12—C11—C5	118.1 (7)	F6A—C36A—F4A	105.6 (9)
C15—C11—C5	125.4 (7)	F5A—C36A—S2A	112.2 (8)
C13—C12—C11	120.9 (6)	F6A—C36A—S2A	111.7 (7)
C13—C12—H12	119.5	F4A—C36A—S2A	106.8 (7)
C11—C12—H12	119.5	O4A—S2A—O6A	117.2 (8)
N3—C13—C12	121.0 (6)	O4A—S2A—O5A	111.6 (6)
N3—C13—H13	119.5	O6A—S2A—O5A	113.6 (8)
C12—C13—H13	119.5	O4A—S2A—C36A	106.8 (6)
C13—N3—C14	120.4 (6)	O6A—S2A—C36A	107.2 (8)
C13—N3—C16	120.1 (5)	O5A—S2A—C36A	98.2 (6)
			
C4—N1—C1—C10^i^	−179.03 (14)	C6—C5—C11A—C15A	73.1 (8)
Ca1—N1—C1—C10^i^	3.8 (2)	C4—C5—C11A—C15A	−108.5 (7)
C4—N1—C1—C2	1.37 (17)	C15A—C11A—C12A—C13A	0.0 (3)
Ca1—N1—C1—C2	−175.84 (10)	C5—C11A—C12A—C13A	−179.5 (11)
C4—N1—C1—Ca1	177.21 (18)	C11A—C12A—C13A—N3A	−0.1 (3)
N1—C1—C2—C3	−1.34 (18)	C12A—C13A—N3A—C14A	0.6 (6)
C10^i^—C1—C2—C3	179.06 (15)	C12A—C13A—N3A—C16A	−179.9 (10)
Ca1—C1—C2—C3	−4.8 (2)	C13A—N3A—C14A—C15A	−0.9 (8)
C1—C2—C3—C4	0.71 (18)	C16A—N3A—C14A—C15A	179.6 (9)
C1—N1—C4—C5	−178.69 (14)	N3A—C14A—C15A—C11A	0.7 (8)
Ca1—N1—C4—C5	−1.4 (2)	C12A—C11A—C15A—C14A	−0.2 (6)
C1—N1—C4—C3	−0.93 (16)	C5—C11A—C15A—C14A	179.3 (10)
Ca1—N1—C4—C3	176.37 (10)	C13A—N3A—C16A—C17A	103.7 (9)
C1—N1—C4—Ca1	−177.29 (17)	C14A—N3A—C16A—C17A	−76.7 (10)
C2—C3—C4—N1	0.11 (18)	N3A—C16A—C17A—C18A	−95.8 (8)
C2—C3—C4—C5	177.93 (15)	N3A—C16A—C17A—C22A	84.5 (8)
C2—C3—C4—Ca1	3.4 (2)	C22A—C17A—C18A—C19A	−0.2 (4)
N1—C4—C5—C6	1.8 (2)	C16A—C17A—C18A—C19A	−179.9 (3)
C3—C4—C5—C6	−175.64 (15)	C17A—C18A—C19A—C20A	−0.2 (6)
Ca1—C4—C5—C6	1.09 (16)	C18A—C19A—C20A—C21A	0.2 (8)
N1—C4—C5—C11A	−176.3 (7)	C19A—C20A—C21A—C22A	0.3 (8)
C3—C4—C5—C11A	6.2 (7)	C20A—C21A—C22A—C17A	−0.8 (8)
Ca1—C4—C5—C11A	−177.1 (7)	C18A—C17A—C22A—C21A	0.8 (6)
N1—C4—C5—C11	−177.6 (4)	C16A—C17A—C22A—C21A	−179.5 (4)
C3—C4—C5—C11	4.9 (5)	C9—C10—C23—C24	60.9 (2)
Ca1—C4—C5—C11	−178.4 (4)	C1^i^—C10—C23—C24	−120.65 (17)
C9—N2—C6—C5	−179.82 (15)	C9—C10—C23—C27	−116.97 (17)
Ca1—N2—C6—C5	5.5 (2)	C1^i^—C10—C23—C27	61.4 (2)
C9—N2—C6—C7	0.13 (17)	C27—C23—C24—C25	4.0 (2)
Ca1—N2—C6—C7	−174.54 (11)	C10—C23—C24—C25	−174.02 (15)
C9—N2—C6—Ca1	174.66 (18)	C26—N4—C25—C24	−1.1 (3)
C4—C5—C6—N2	−4.2 (3)	C28—N4—C25—C24	177.82 (16)
C11A—C5—C6—N2	174.0 (7)	C23—C24—C25—N4	−2.1 (3)
C11—C5—C6—N2	175.3 (4)	C25—N4—C26—C27	2.3 (3)
C4—C5—C6—C7	175.87 (16)	C28—N4—C26—C27	−176.68 (16)
C11A—C5—C6—C7	−6.0 (7)	N4—C26—C27—C23	−0.2 (3)
C11—C5—C6—C7	−4.7 (5)	C24—C23—C27—C26	−2.8 (3)
C4—C5—C6—Ca1	−1.08 (16)	C10—C23—C27—C26	175.14 (16)
C11A—C5—C6—Ca1	177.1 (7)	C25—N4—C28—C29	93.82 (19)
C11—C5—C6—Ca1	178.4 (4)	C26—N4—C28—C29	−87.2 (2)
N2—C6—C7—C8	0.26 (19)	N4—C28—C29—C34	−17.9 (2)
C5—C6—C7—C8	−179.80 (16)	N4—C28—C29—C30	164.71 (16)
Ca1—C6—C7—C8	−4.6 (2)	C34—C29—C30—C31	0.5 (3)
C6—C7—C8—C9	−0.5 (2)	C28—C29—C30—C31	178.00 (18)
C6—N2—C9—C10	−178.49 (15)	C29—C30—C31—C32	−0.6 (3)
Ca1—N2—C9—C10	−4.0 (2)	C30—C31—C32—C33	0.0 (3)
C6—N2—C9—C8	−0.44 (17)	C31—C32—C33—C34	0.8 (3)
Ca1—N2—C9—C8	174.08 (11)	C30—C29—C34—C33	0.3 (3)
C6—N2—C9—Ca1	−174.52 (19)	C28—C29—C34—C33	−177.04 (18)
C7—C8—C9—N2	0.60 (19)	C32—C33—C34—C29	−0.9 (3)
C7—C8—C9—C10	178.62 (16)	F1—C35—S1—O3	−69.7 (7)
C7—C8—C9—Ca1	5.6 (2)	F2—C35—S1—O3	53.1 (7)
N2—C9—C10—C1^i^	1.6 (3)	F3—C35—S1—O3	171.7 (6)
C8—C9—C10—C1^i^	−176.11 (16)	F1—C35—S1—O2	52.1 (7)
Ca1—C9—C10—C1^i^	−0.62 (16)	F2—C35—S1—O2	174.8 (6)
N2—C9—C10—C23	179.82 (14)	F3—C35—S1—O2	−66.6 (6)
C8—C9—C10—C23	2.2 (2)	F1—C35—S1—O1	173.8 (6)
Ca1—C9—C10—C23	177.66 (11)	F2—C35—S1—O1	−63.5 (7)
C6—C5—C11—C12	−95.2 (5)	F3—C35—S1—O1	55.1 (6)
C4—C5—C11—C12	84.3 (5)	F3A—C35A—S1A—O1A	64.2 (9)
C6—C5—C11—C15	86.8 (6)	F2A—C35A—S1A—O1A	−57.0 (9)
C4—C5—C11—C15	−93.7 (6)	F1A—C35A—S1A—O1A	−176.5 (7)
C15—C11—C12—C13	−0.4 (2)	F3A—C35A—S1A—O3A	−171.9 (8)
C5—C11—C12—C13	−178.7 (6)	F2A—C35A—S1A—O3A	66.8 (9)
C11—C12—C13—N3	−0.6 (3)	F1A—C35A—S1A—O3A	−52.7 (9)
C12—C13—N3—C14	1.5 (5)	F3A—C35A—S1A—O2A	−59.5 (10)
C12—C13—N3—C16	−173.5 (6)	F2A—C35A—S1A—O2A	179.3 (8)
C13—N3—C14—C15	−1.3 (6)	F1A—C35A—S1A—O2A	59.8 (9)
C16—N3—C14—C15	173.8 (6)	F6—C36—S2—O6	−58.2 (7)
N3—C14—C15—C11	0.2 (6)	F5—C36—S2—O6	−171.1 (8)
C12—C11—C15—C14	0.6 (5)	F4—C36—S2—O6	68.1 (9)
C5—C11—C15—C14	178.7 (7)	F6—C36—S2—O5	61.3 (7)
C13—N3—C16—C17	101.8 (6)	F5—C36—S2—O5	−51.6 (8)
C14—N3—C16—C17	−73.2 (6)	F4—C36—S2—O5	−172.4 (8)
N3—C16—C17—C22	94.9 (5)	F6—C36—S2—O4	179.7 (8)
N3—C16—C17—C18	−84.8 (4)	F5—C36—S2—O4	66.9 (9)
C22—C17—C18—C19	−0.2 (3)	F4—C36—S2—O4	−54.0 (10)
C16—C17—C18—C19	179.5 (2)	F5A—C36A—S2A—O4A	−83.9 (9)
C17—C18—C19—C20	0.6 (5)	F6A—C36A—S2A—O4A	52.2 (10)
C18—C19—C20—C21	0.1 (7)	F4A—C36A—S2A—O4A	167.3 (8)
C19—C20—C21—C22	−1.3 (7)	F5A—C36A—S2A—O6A	42.6 (11)
C20—C21—C22—C17	1.8 (7)	F6A—C36A—S2A—O6A	178.7 (10)
C18—C17—C22—C21	−1.0 (5)	F4A—C36A—S2A—O6A	−66.3 (10)
C16—C17—C22—C21	179.3 (3)	F5A—C36A—S2A—O5A	160.5 (8)
C6—C5—C11A—C12A	−107.4 (8)	F6A—C36A—S2A—O5A	−63.4 (9)
C4—C5—C11A—C12A	70.9 (8)	F4A—C36A—S2A—O5A	51.6 (8)

Symmetry code: (i) −*x*+1, −*y*+2, −*z*+1.

### Hydrogen-bond geometry (Å, º)

**Table:**

*D*—H···*A*	*D*—H	H···*A*	*D*···*A*	*D*—H···*A*
O7—H7*A*···O1	0.86 (2)	2.04 (2)	2.817 (5)	150 (3)
O7—H7*A*···O3*A*	0.86 (2)	2.14 (2)	2.874 (6)	143 (3)
O7—H7*B*···O4	0.88 (2)	2.24 (3)	2.965 (11)	140 (3)
O7—H7*B*···O6*A*	0.88 (2)	2.29 (3)	3.048 (10)	144 (3)
O8—H8*A*···O2^ii^	0.87 (2)	2.08 (2)	2.914 (6)	163 (3)
O8—H8*A*···O2*A*^ii^	0.87 (2)	1.94 (2)	2.789 (7)	167 (3)
O8—H8*B*···O7	0.89 (2)	1.96 (2)	2.838 (3)	174 (3)

Symmetry code: (ii) −*x*+2, *y*−1/2, −*z*+3/2.
